# Fractures diagnosed in primary care – a five-year retrospective observational study from a Norwegian rural municipality with a ski resort

**DOI:** 10.1080/02813432.2019.1685202

**Published:** 2019-11-13

**Authors:** Stein Vabo, Knut Steen, Christina Brudvik, Steinar Hunskaar, Tone Morken

**Affiliations:** a Vennesla Health Care Center, Vennesla, Norway;; b National Centre for Emergency Primary Health Care, NORCE Norwegian Research Centre, Bergen, Norway;; c Department of Clinical Medicine, University of Bergen, Bergen, Norway;; d Department of Global Public Health and Primary Care, University of Bergen, Bergen, Norway

**Keywords:** Epidemiology, musculo-skeletal conditions, family practice, diagnostic methods, health services research

## Abstract

**Objective:** The aim of this study was to characterize fractures recorded at a Norwegian primary care centre near a ski resort.

**Design:** A retrospective five-year observational study in the period 2010–2014.

**Setting:** A primary care centre equipped with an x-ray machine and located near a ski resort in a small rural municipality of 931 inhabitants in Norway. The X-ray images are digitalized and instantly transferred for assessment of a radiologist and/or an orthopedic surgeon both before and after treatment.

**Subjects:** All patients with radiologically confirmed fractures.

**Results:** A total of 1154 X-ray examinations were done, out of which 480 (41.6%) were fractures verified by a radiologist. The most frequent fractures were in the wrist (30%), collarbone (15%), shin (11%), humerus (9%) and ankle (8%). 316 (66%) of the fractures were in males and of these 225 were in age group 10–19 years. Males dominated among fractures in collarbone (92% males), finger (80% males), and foot (85% males). Women with fractures of the wrist, ankle, humerus and metacarpal bones, had a higher median age than men with similar fractures. Nonsurgical treatment with cast or braces was initially offered in 371 (77%) of the fracture-cases at the primary care level.

**Conclusion:** Young men acquired most of the fractures, predominantly in the wrist, and mostly during the winter sport season. Nearly eight of ten fractures were treated locally in primary care centre.

**Key points**
- A large seasonal variation was found in number of patients with fractures.
- More than 60% had fractures in the wrist, collarbone, shin or ankle.
- More than half of the patients with a fracture were males and below 20 years old.
- Most fractures were ski-related.

## Introduction

In Norway, primary health care has the main responsibility for all medical emergencies, including traumas [1]. All admissions to hospital care and specialist services, including radiology, are principally by referral after an initial primary care assessment. Radiology services are thus usually not directly available to general practitioners and doctors in «out-of-hours» services. If X-ray is indicated, the patient is referred to a department of radiology, either at a hospital or at an outpatient unit.

However, some Norwegian municipalities or out-of-hours services have invested in X-ray facilities, mostly due to remoteness of the nearest radiological department, often combined with a high volume of injuries requiring imaging. These municipalities are usually large ski resorts and most of the fractures diagnosed are caused by ski related injuries.

Many epidemiological studies of injuries at ski resorts have identified knee injuries, with or without fractures, as the most frequent injury in alpine skiing activities [2], whereas snowboarders sustain more injuries to the upper limb and axial areas [3]. The data in these studies have been collected from first aid or emergency services at the ski resort without radiological services available for diagnostic use near the injury site [4–6]. Previous studies have mainly described the epidemiology of all types of injuries and have not been limited to only fractures [2–6]. Some hospital-based studies have described fractures occurring in ski resorts [7,8]. However, studies from a rural primary care setting, covering the whole range of traumatic fractures of varied severity, are scarce.

The aim of this study was to investigate the radiologically diagnosed fractures at a primary care centre equipped with X-ray facilities in a Norwegian remote rural municipality with a ski resort.

## Material and methods

We performed a retrospective observational study covering five years from 2010 to 2014. All patients with X-ray verified fractures, diagnosed at the primary care centre of Bykle, were included. The radiological department at the regional hospital in Arendal verified the fracture diagnoses.

### Setting

The population of Bykle municipality was 931 in 2014, but a large number of tourists visit the area all year round, especially during winter and early spring. The village of Hovden, located in the municipality, is the largest ski resort in the most southern part of Norway. Ranked by number of visitors, it is the fifth-largest ski resort in Norway. The main tourist season is from mid-December to late April. During Easter, there may be as many as 20,000 people in the area daily. Due to the large number of fractures caused by the winter sports activities, the primary care centre in Bykle has offered locally operated radiology services since 1988. From 2004 the X-ray images were digitalized. There is only one primary care centre in the municipality. This single centre is responsible for providing primary health care at any time for all people who at the time of the accident are located in the municipality. In the busiest weekends during the winter and during the winter holidays, the number of doctors and nurses on duty are doubled.

The travelling distance to the nearest hospital with orthopaedic clinic and X-ray laboratory is 200 km, three hours travelling by car or ambulance.

When someone is injured in Bykle, the local emergency call centre is contacted, and the person is transported to the primary care centre by private car, taxi, or ambulance. The most severely injured patients are transported directly to hospital by ambulance or helicopter. These patients sometimes need medical examination and stabilization by the GP before further transport, but some will bypass the primary care physician on call.

The physician on call first examines the patient at the primary care clinic. If imaging is found necessary, the X-ray examination is performed by the same physician or by a locally trained nurse. The X-ray images are digitalized and instantly transferred for assessment both before and after treatment. Besides his/her own basic knowledge in radiological diagnostics, the physician on call receives an almost instant professional radiological assessment by the radiologist or orthopaedic surgeon on call at the regional hospital. The X-ray examinations and fracture treatments are in accordance with recommended and updated procedures and based on written guidelines [9,10].

### Data sources

The X-ray reports from the radiologists were included in the medical records of the patients. For purpose of this study, the first author (SV) reviewed the X-ray archive and the medical records in order to identify all patients with fractures. The following information was recorded from the X-ray archive: Age, sex, type of fracture and date of injury. Information recorded from the medical records included a description of fracture from the radiologist, type of activity (ski-related or not), place of treatment (locally or hospital), resident in the municipality or not. To establish an estimate of the female/male-ratio at the ski resort, we performed a manual count at the main ski lift on the sex of the skiers. The first author counted all persons who entered the ski lift during two hours.

### Statistics

The results are presented as frequencies with absolute numbers and percentages. Distributions of categorical variables are compared between groups with chi-square tests. Due to skewed distribution of age, Mann–Whitney *U* Test was used to test for difference in age with respect to sex. Statistical significance was set at *p* < .05. The software program SPSS version 23 was used for statistical analyses.

The project was approved by the Regional Committee for Medical and Health Research Ethics (approval no. 2015/59).

## Results

In the period 2010–2014, the primary care centre performed 1154 X-ray examinations after trauma. Among these, 480 (41.6%) were diagnosed with fractures by a radiologist. In addition, eleven patients with serious injuries and possible fractures, were brought directly to hospital without prior medical assessment including radiological diagnostics at the primary care centre. These patients were not included in the study.

**Figure 1.**
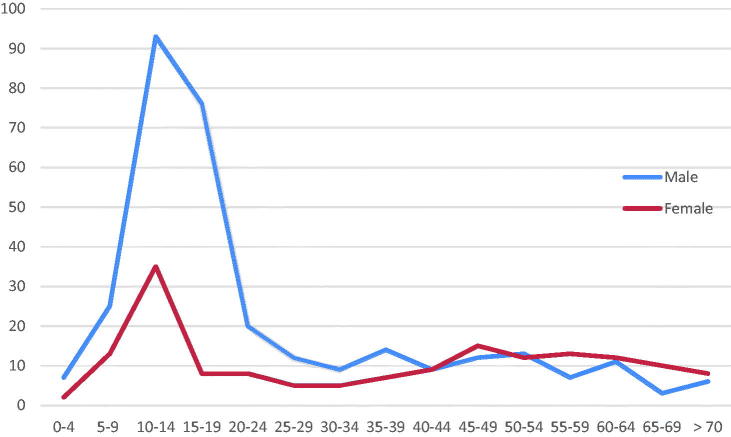
The distribution of patients with fractures by sex and age groups (*n* = 480).

Of the 480 patients with radiologically diagnosed fractures, 316 were males (65.8%). The average age was 28.1 years (median = 17.0 years, range from 1 to 94 years, see Figure 1.

**Figure 2.**
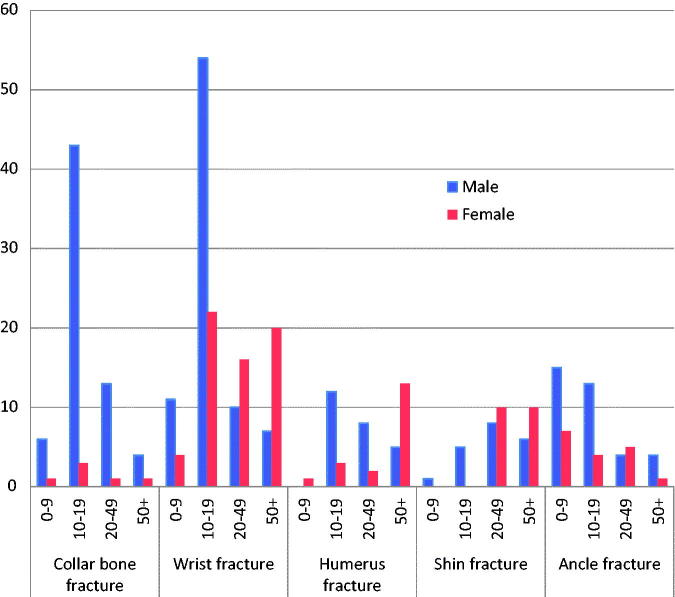
Number of patients with the five most common fractures by sex and age groups (*n* = 353).

Wrist fracture was most frequent (30.0%), followed by collarbone fracture (15.0%), shin fracture (11.0%), humerus fracture (9.2%) and ankle fracture (8.3%) (Table 1). The patients with these four types of fracture comprised 73.5% of all patients with fractures (Figure 2). 53.2% of the male patients with fractures were in the age group between 10 and 19 years, but only 13.9% of the female patients were in this young age group. In the older age group over 50 years, women predominated among the fracture patients with 34.1%, while 23.8% were men.

**Table 1.** Patients with fractures diagnosed with local radiology 2010–2014.

Type of fracture	All					Male					Female				
	*n*	(% of all fractures)	Age Median	Age, mean	(SD)	*n*	(% of males within fracture type)	AGE Median	Age, mean	(SD)	*n*	(% of females within fracture type)	AGE Median	Age, mean	(SD)
Wrist	144	(30.0)	15.0	25.4	(19.9)	81	(56.3)	14.0	18.3	(14.6)	63	(43.8)	30.0	34.4	(22.2)
Collarbone	72	(15.0)	16.0	21.0	(13.7)	66	(91.7)	16.0	20.7	(13.0)	6	(8.3)	12.5	24.5	(21.7)
Shin (tibia, fibula)	53	(11.0)	13.0	19.5	(18.0)	32	(60.4)	12.0	18.5	(18.1)	21	(39.6)	14.0	21.0	(18.2)
Ankle	40	(8.3)	46.0	41.6	(17.3)	21	(52.5)	32.0	34.5	(18.6)	19	(47.5)	50.0	49.4	(11.9)
Finger	39	(8.1)	16.0	22.7	(15.0)	31	(79.5)	17.0	24.5	(16.0)	8	(20.5)	12.0	16.1	(8.1)
Humerus Prox. + Dist.	44	(9.2)	42.0	38.3	(22.4)	25	(56.8)	21.0	29.5	(17.1)	19	(43.2)	58.0	55.6	(20.1)
Metacarpal	33	(6.9)	22.0	31.4	(21.4)	21	(63.6)	16.0	21.5	(12.8)	12	(36.4)	44.0	48.7	(23.0)
Foot	20	(4.2)	37.5	39.9	(20.5)	17	(85.0)	37.0	39.1	(21.9)	3	(15.0)	44.0	44.0	(11.5)
Other upper extremities	18	(3.8)	27.5	32.7	(19.6)	12	(66.7)	24.5	34.1	(21.1)	6	(33.3)	31.5	30.0	(17.5)
Other lower extremities	16	(3.3)	28.5	38.0	(29.4)	9	(53.8)	23.0	32.9	(24.6)	7	(46.2)	34.0	44.7	(17.5)
Columna	1	(0.2)	–	–	–	1	–	–	–	–	1	–	–	–	–
Total fractures	480	(100)	17.0	28.1	(20.3)	316	(65.8)	16.0	23.8	(17.39)	164	(34.2)	36.5	36.4	(22.9)

### Wrist fractures

Women with wrist fractures were significantly older than men with this fracture (*p* < .001). More than half of the patients with wrist fractures (52.3%) were in the young age group (10–19 years), and 71.1% of them were males. 18.1% of patients with wrist fractures were in the older age group over 50 years and 76.9% were females.

### Collarbone fractures

More males than females had a collarbone fracture (*p* < .001), but there was no significant age difference between the sexes. Among the patients with collarbone fractures, 63.9% were in the age group 10–19 years, and they were nearly all males. (93.5%).

### Shin fractures

Of the 53 patients with shin fractures, 69.8% were younger than 20 years, and 64.9% were males.

### Ankle fractures

We found no significant sex difference in mean age, among patients with ankle fractures. Most patients (87.5%) were over 20 years of age, and no women with ankle fractures were younger than 20 years.

### Finger, foot, proximal humeral bone and metacarpal fractures

Among the patients with all the other categories of fracture, we found significantly more men with fractures of fingers (*p* < .001) and feet (*p* < .001). Women were significantly older than men among patients with fractures of the proximal humerus (*p* < .001) and the metacarpal bones (*p* < .001).

### Seasonal variations and skiing/snowboard activities

**Figure 3.**
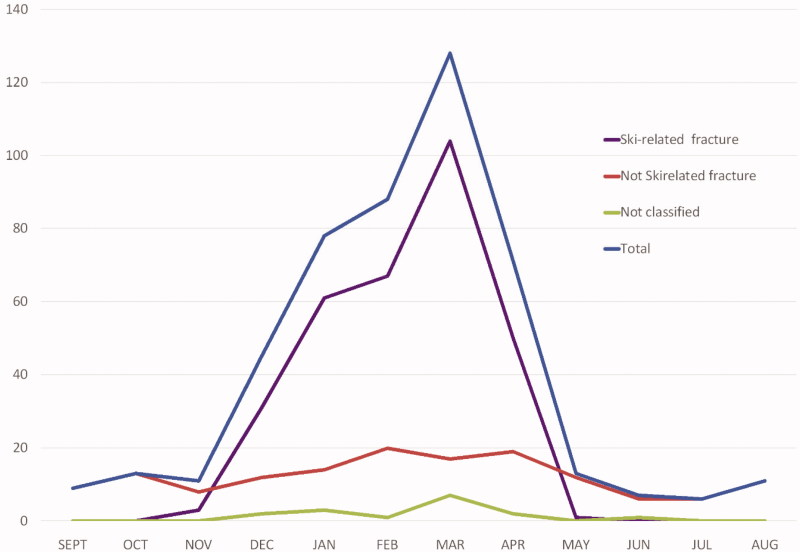
Seasonal variation in patients with fractures by month of the year 2010–2014 (*n* = 480).

Figure 3 shows the seasonal variation of all patients with fractures, and the proportion that occurred during skiing/snowboarding activities and those unrelated to these activities. Most fractures (84.4%) occurred during the skiing season, during the winter months and in spring (December-April), and they were often ski/snowboard-related. Of the total of 480 fractures, 66.0% were described as ski/snowboard related injuries; 30.6% were not ski/snowboard related and in 3.3% the type of activity leading to the injury was not described.

### Male/female distribution and the amount of tourists

Among 1285 people taking the ski lift during two hours in December 2018, we found a sex distribution of 703 men (54.7%) and 582 women (45.3%)

Information from the medical records showed that 414 (86%) of the fracture patients were tourists, and only 66 (14%) patient were living in the municipality.

### Medical treatment

Initial nonsurgical treatment with casts or braces was initially offered at the primary care level to 77.3% of the patients with fractures (*n* = 371), and 22.7% of the patients were transported to hospital.

## Discussion

This study of 480 patients with fractures diagnosed in primary care in a Norwegian rural municipality with a ski resort in the period 2010–2014 shows that fractures of the wrist, collarbone, humerus, shin and ankle were the most frequent fractures. Most of the fractures were registered among boys and young men aged 10 to19 years, and during the winter sport season. The study also shows that most of the patients were tourists and most of the fractures were associated with skiing and snowboarding activities. We think that the clinical diagnostic precision prior to the X-rays seems quite acceptable as more than 40 percent of the radiological images were identified as a fracture during the period 2010–2014. This corresponds well with a study from the Netherlands showing 41.3% of x-rays in primary care were positive of a fracture [11]. This may indicate that the threshold for referral to X-ray examination based on clinical assessment is restrictive in primary health care in both countries, and acceptable regarding radiation hygiene. The Dutch study also found that the threshold for requesting X-rays increases with increasing distance to the X-ray facility, and with a relatively high number of initially undiagnosed fractures (13.6%) as a consequence [11]. It is possible that without local X-ray facilities this would also apply to the municipality of Bykle. A distance of 200 km to the nearest hospital, often over snowy and icy roads in wintertime, increases the threshold for patient transportation, with an increased risk of missed fracture diagnoses.

The history of conservative treatment of fractures in the municipality of Bykle started in 1988, when ordinary X-ray was introduced to the primary care. In 2004, when digitalisation of X-rays was introduced, the images could be instantly transferred to the radiologist or orthopaedic surgeon at the hospital for assessment. It was a tremendous improvement for the GPs to be able to consult instantly with hospital specialists about both diagnosis and recommended treatment. For the years 2010–2014, an average of 77.3% of the diagnosed fractures were initially treated at the primary care centre.

Wrist fracture was the most common fracture in our study as nearly one-third of all patients had this type of fracture. This is a larger proportion than found in population-based epidemiological studies of adults [12–14] where the percentages vary from 17.5 to 20. Our findings correspond better to an all paediatric population, where this type of fracture accounts for 25% to 30% [15,16]. We assume that a high proportion of children in our study population were exposed to winter sport activities with a high risk of wrist injury [17].

Collarbone fracture was the second most frequent fracture in this study, and twice the proportion seen in child population studies [13,15]. Even this type of fracture is characteristic of children injured in sports related activities. Likewise, shin fractures occurred twice as often (11.0%) as compared to other studies [13,15]. We think that skiing activities can explain the high proportions of patients with fractures of wrist, collarbone and shin in our study. We suppose that skiing and snowboarding activities in the slopes, with high speed and jumping activities, increase the risk of this kind of injuries [18]. There were no registered ankle fractures among females younger than 20 years of age, and only a few among males. This is somewhat different from epidemiological studies on fractures where ankle fractures are quite common in children and young people [15]. In adults, our study showed the same female/male distribution as other studies [13–15], with a majority of fractures in young men. However, after 50 years of age, we found a shift to more women than men with fractured ankles [13,14]. We had expected that high-energy activities like slalom and snowboarding would cause a high number of ankle fractures. The low frequency of ankle fractures might be due to improved equipment used for skiing in the slopes [19]. Snowboard and slalom boots are rigid and protective around the ankle joint and are supposed to prevent injury at this site [19–21]. Ankle fractures were more common in the age group above 50 years, possibly because cross-country skiing is the preferred activity in this older age group. The traditional boots for this skiing activity give less stability and protection to the ankle.

In general, male patients with fractures were significantly younger than female patients. Most of the patients with fractures in collarbone, fingers or feet were boys or young men aged 10 to 19 years. They had nearly four times as many fractures as girls and young women at the same age group. This difference can probably not be explained by more male than female skiers in the slopes as the manual count showed only a slight predominance of males in the slope (55% versus 45%). We think this count is representative for the sex distribution of the total population in the slopes. The sex and age differences are supported by several epidemiological studies of fractures [12,13,20]. It is well known that boys have a generally higher risk behavior than girls, in both sports and leisure activities [15]. Skiing and especially slalom and snowboard invite to behavior with a higher energy impact and a higher risk of injury. However, we do not know the male/female ratio for the population at risk in this age group.

Among females, the proportion of patients with fractures seemed to increase with age. Fractures of the wrist, ankle, humerus and metacarpal bones were more frequent among women older than 50 years of age. Other studies confirm this shift in increased risk of fracture from in the mid-fifties, and it has been explained by a higher incidence of osteoporosis after menopause [12,13,22]. This relatively higher age among females is also found in other studies of fracture distributions [12,13,22].

### Seasonal variation

We found a large seasonal variation in number of patients with fractures, as found elsewhere [23]. A study from Wales on wrist fractures found an increase in the winter months for people aged over 75, but a decrease in the winter for children under the age of 15 [24]. Other studies show that most of the fractures occur in the winter season and that snow and ice seem to contribute [23,25]. In Bykle there is normally ice and snow for more than six months each year. The large seasonal variation of fractures may be explained by winter sports and corresponds well with the fact that most of the injuries are ski-related and therefore occur during the winter. In addition, generally more people stay in the municipality during winter than during summer.

### Medical treatment

Nonsurgical treatment with casts or braces was initially offered at the primary care level in 77.3% of the fractures. The introduction of digitalized x-ray examinations in a small municipality such as Bykle, with a long distance to the nearest hospital has several effects.

First, diagnosing and treating the patient in primary care may reduce number of long and unnecessary trips to the hospital. This would probably save time and money, for both the patient and society.

Secondly, avoiding ambulance for transportation of patients with possible fractures will maintain the emergency preparedness in the municipality. Lastly, diagnosing and treating fractures in general practice will improve the emergency medical professionalism in the municipality's health service.

In a Dutch study from primary health care 66.4% were initially treated locally [11]. The explanation for the higher proportion of treated patients in our Norwegian study, could be more experience in treating fractures in rural GP practice in general, and specifically a longer tradition in our municipality in treating fractures. Another explanation could be different distribution of fracture types in the studies.

### Workload at the primary care centre

The tourist activities in the winter season (weekends, Christmas, winter-holiday and Easter) induce a large workload for the physicians and nurses in Bykle. In order to cope with the increased number of injuries and fractures, the number of doctors and nurses on the busiest days is doubled.

### Strengths and limitations

A strength of the study is that nearly all the patients staying in the municipality at the time of injury, were included. Eleven patients with serious injuries and possible fractures were transported directly to hospital and these patients are not included in the study. In addition some other patients with possible fractures may have received treatment at their home place thus bypassed the out-of-hours service in Bykle. However, due to long travelling distance (more than two hours of transport time from the ski resort to the next general practitioner office), we think these cases are probably few. We, therefore, assume that almost all fractures from injuries that occurred in the municipality in the study period were registered and included in the study. It is also a strength of the study that the diagnosis of fractures, were validated and confirmed by a specialist in radiology.

A limitation of the study is the lack of accurate information about the number, age and sex of people staying in the municipality at each point in time, and we were, therefore, unable to estimate incidence rates. Also, we did not have information about the number of hours the tourists spent in the slopes in order to calculate the risk of fracture by time performing different winter sport activities.

## Conclusions

This five-year retrospective observational study illustrates the fracture pattern in a remote rural community with a ski resort. We found most fractures in the wrist, collarbone, shin or ankle, mostly among young males during the winter sport season. Most fractures are ski-related during the wintertime. Non-ski-related fractures also seem to be more common in wintertime. Nearly 80% of the diagnosed fractures were initially treated by primary health care. The clinical workload for physicians and nurses at the primary care centre increases significantly during the winter season.

## Implications

The study adds valuable knowledge about the fracture pattern in a rural municipality with a ski resort. The quality and costs of radiological examinations, diagnosis and treatment of fractures in the primary health care setting should be further investigated.
